# Promising improvement in infected Wound Healing in Type two Diabetic rats by Combined effects of conditioned medium of human adipose-derived stem cells plus Photobiomodulation

**DOI:** 10.1186/s42826-023-00178-z

**Published:** 2023-11-15

**Authors:** Kaysan Sohrabi, Houssein Ahmadi, Abdollah Amini, Behnaz Ahrabi, Atarodalsadat Mostafavinia, Hamidreza Omidi, Mansooreh Mirzaei, Fatemeh Fadaei Fathabady, Mohammadjavad Fridoni, Maryam Rahmannia, Sufan Chien, Mohammad Bayat

**Affiliations:** 1 School of Medicine, Shahid Beheshti University of Medical Sciences, Tehran, Iran; 2 Department of Biology and Anatomical Sciences, Shahid Beheshti University of Medical Sciences, Tehran, Iran; 3 Department of Anatomical Sciences and Cognitive Neuroscience, School of Medicine, Tehran Medical Sciences, Islamic Azad University, Tehran, Iran; 4 Department of Anatomy, School of Medicine, Mazandaran University of Medical Sciences, Sari, Iran; 5 Department of Biology and Anatomical Sciences, School of Medicine, Zanjan University of Medical Sciences, Zanjan, Iran; 6 Price Institute of Surgical Research, University of Louisville and Noveratech LLC, Louisville, KY USA; 7 Department of Biology and Anatomical Sciences, School of Medicine, Shahid Beheshti University of Medical Sciences, Tehran, Iran

**Keywords:** Diabetes mellitus, Diabetic foot ulcers, Human adipose-derived stem cell, Conditioned medium, Photobiomodulation, Microbial flora, Stereology, Rat

## Abstract

**Background:**

We aimed to examine the accompanying and solo impacts of conditioned medium of human adipose-derived stem cells (h-ASC-COM) and photobiomodulation (PBM) on the maturation stage of an ischemic infected delayed-healing wound model (IIDHWM) of rats with type 2 diabetes (TIIDM).

**Results:**

Outcomes of the wound closure ratio (WCR) results, tensiometrical microbiological, and stereological assessment followed almost identical patterns. While the outcomes of h-ASC-COM + PBM, PBM only, and h-ASC-COM only regimes were significantly better for all evaluated methods than those of group 1(all, *p* < 0.001), PBM alone and h-ASC-COM + PBM therapy achieved superior results than h-ASC-COM only (ranged from *p* = 0.05 to *p* < 0.001). In terms of tensiometrical and stereological examinations, the results of h-ASC-COM + PBM experienced better results than the PBM only (all, *p* < *0.001*).

**Conclusions:**

h-ASC-COM + PBM, PBM, and h-ASC-COM cures expressively accelerated the maturation stage in the wound healing process of IIDHWM with MRSA in TIIDM rats by diminishing the inflammatory reaction, and the microbial flora of MRSA; and increasing wound strength, WCR, number of fibroblasts, and new blood vessels. While the h-ASC-COM + PBM and PBM were more suitable than the effect of h-ASC-COM, the results of h-ASC-COM + PBM were superior to PBM only.

**Supplementary Information:**

The online version contains supplementary material available at 10.1186/s42826-023-00178-z.

## Background

Type 2 diabetes mellitus (TII DM) is caused by a gradual loss of βeta-cell’s ability for insulin secretion sufficiently, almost in the presence of insulin resistance [1].

The increasing prevalence of diabetes mellitus (D.M.) worldwide has remained a remarkable public health worry, creating overwhelming demands on people, healthcare systems, caregivers, and society at large. D.M. is mainly categorized into two principal types: type 1 diabetes mellitus (TI DM) and TIIDM. TIIDM has a higher rate of prevalence, accounting for more than 85% of all cases of D.M. [2]. TIIDM is one of the most globally challenging and prevalent metabolic disorders affecting 10.5% of adults [3]. 11.3 percent, or 37.3 million U.S. residents, suffer from D.M. Moreover, individuals with preDM are at increased risk of developing TIIDM, chronic kidney disease, and cardiovascular disease. There are 1 in 3 adults in the U. S. (96 million) who have preD.M. Of those 96 million, more than 76.8 million don’t even know they have it. Without action, many people with preDM could develop TIIDM within 5 years [4]. Diabetic foot disease (DFD) is counted as a familiar complication of D. M. that is particularly concerning. The most terrible consequence of DFD is amputation, which may cause patients great physical and emotional distress and substantial economic costs. Diabetic foot ulcers (DFU) s often precede the requirement for amputation [5].

DFUs are probable to be developed due to neuropathy, ischemia, or a mix of them, and when the infection gets on the DFU, this mixture can become limb and life-threatening [5]. DFU, the most common complication of D.M., is observed in up to 34% of diabetic patients [6]. The total medical cost for managing DFD in the U. S. is about US$11 billion per year [7]. Despite these excessive financial costs, roughly 20 percentage of D.M. individuals have remained untreated DFUs during the first year of disease [8]. Existing management for DFUs are unacceptable due to the enhanced danger of bacterial pollution and destructing the process of angiogenesis in wound healing (restoration) [9]. Therefore there is a potential for improved treatment of DFUs, and further investigations are required to discover new approaches to healing severe cases of DFUs [10].

The usage of regenerative medicine and stem cell-based therapeutic methods demonstrated much potential in improving the healing process of wounds. Plastic surgeons have been exploring the practical and clinical application of human adipose-derived stem cells (h-ASCs), which are effortless to obtain adult stem cells that lead to differentiation into multiple types of cells and release growth factors that ease wound restoration. These growth factors assist the process of angiogenesis, leading to an enhancement in local blood flow, as overall result, enhancing the final wound healing process [11].

Additional benefits of h-ASCs can be counted as their ability to be easily obtained in large quantities via simple liposuction surgical procedures, they are capable of maintaining their properties even after long-lasting periods of in vitro cultivation, and their low immunogenicity, which let for the usage of h-ASCs from different donors. It has been proposed that the wound-healing effects of h-ASCs may be associated with reducing inflammation, encouraging blood vessel growth, and boosting the growth of fibroblasts and keratinocytes [12].

Despite extensive investigations on h-ASCs proposed in animal models both in vitro and in vivo, a scarcity of randomized clinical trials presents involving humans. Moreover, the few clinical trials that have been started are still ongoing or in the process of recruiting patients [11].

Although h-ASCs are a good cell type for MSC transplantation therapeutic method in wound healing, patients with D.M. can experience remarkable inhibition of stem cell activity because of D.M.-induced glucolipotoxicity [13], and poor cell survival within the harsh wound microenvironment [14]. The activating of keratinocytes mostly settles the wound restoration effect of ASCs and also dermal fibroblasts by the mechanism called paracrine [15]. De Gregorio et al. [16] distinguished that the application of conditioned medium (COM) obtained from h-ASCs (h-ASC-COM) to diabetic rats obviously regresses the creation of diabetic polyneuropathy (DPN), which enhances the wound restoration process in diabetic mice [16].

Since more than 100 known physiologic factors contribute to wound restoration deficiencies in individuals with D.M. [17], previous studies have shown that using two therapeutic agents concurrently, such as ASC and photobiomodulation(PBM) [18], bone-marrow-derived mesenchymal stem cell‐conditioned medium (BM-MSC-COM) and PBM[19]; metformin and PBM[20]; or a nanoparticle plus PBM [21] could progress more wound restoration in diabetic subjects with TIDM, TIIDM, and healthy subjects through an additive effect of two agents compared to one agent.

PBM is a probably advantageous modality for hastening wound restoration, relief of pain, and decreasing inflammation with modification of different biological processes [22]. Results of three recent studies have shown that a combination of ASCs-COM and PBM significantly improved the restoration process in wounds, flaps, and ischemic wounds of healthy animals [23–25].

In 2012, Montero-Vilchez, in their review article, concluded that MSC-COM is an optimistic therapeutic method for cutaneous problems. Investigations on MSC-COM treatment in animals demonstrated high wound closure rates and acceptable reports of skin rejuvenation. However, more trials are demanded to reassure the safety and efficacy of COM manufacturing [26]. We aimed to examine the accompanying and solo impacts of h-ASC-COM and PBM on the maturation phase of an ischemic infected delayed-restoration wound model (IIDHWM) of TIIDM rats.

## Results

### Marker expression

Flow cytometry demonstrated that the h-ASCs expressed cluster of differentiations (C.D).s 11b 45, 0.33% and 0.8%, respectively. In addition, h-ASC completely expressed C.D.s 44 and 105.

### Gross information

**Fig. 1:**
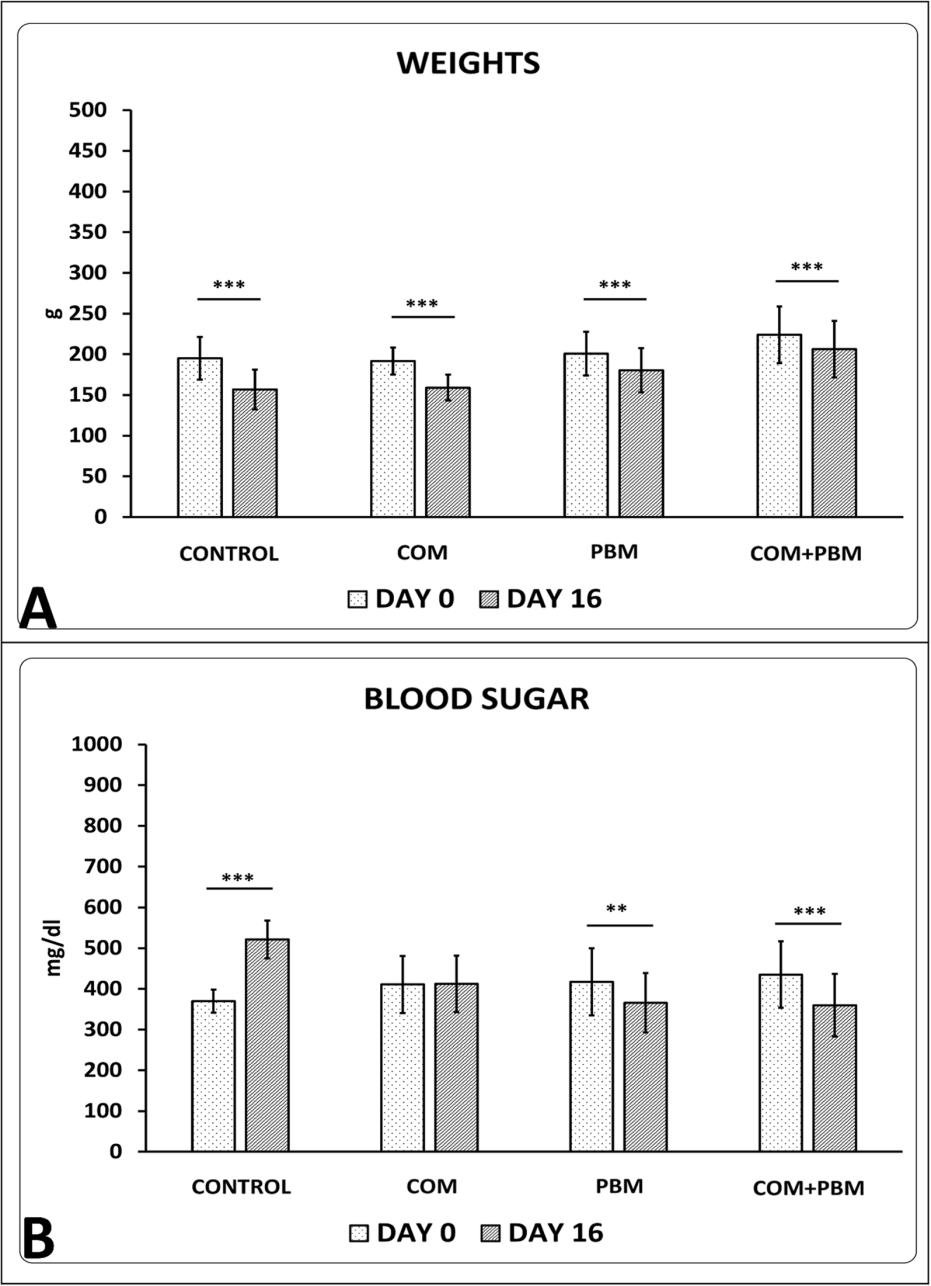
Mean ± S.D. of body weights (**A**) and blood sugar values **B** at the beginning of the study (day 0) and time of sampling (day 16) of studied groups compared by t-test. **p < 0.01; ***p < 0.001

Our observations confirmed significant decreases in weight and blood sugar levels in almost all studied groups (Fig. 1).

### Microbial findings, day 16

**Fig. 2:**
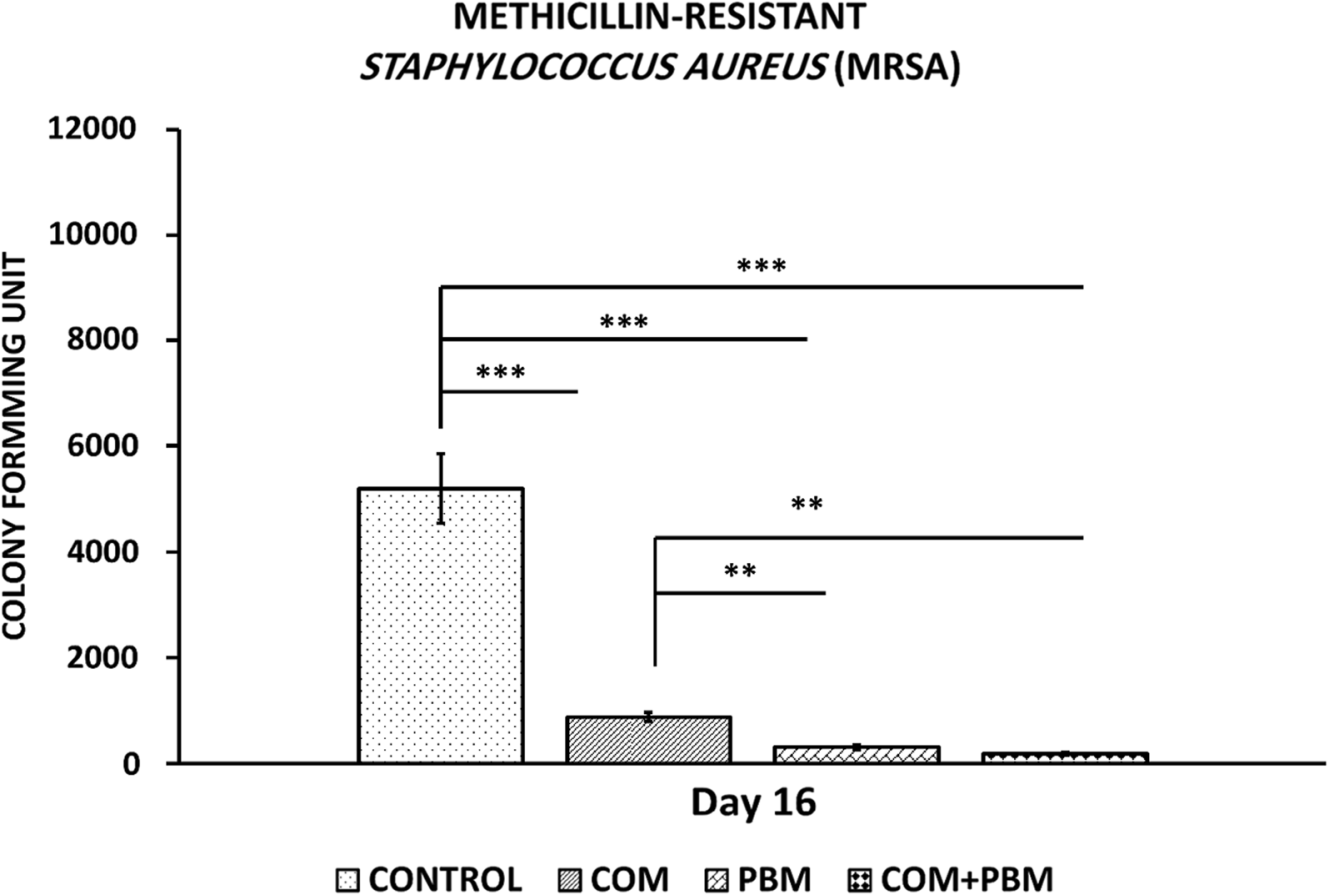
Mean ± S.D. of colony forming units of methicillin-resistant *Staphylococcus aureus* (MRSA) of wounds in each group on day 16.; Mean ± S.D. of studied groups in comparison using ANOVA and LSD tests. *p < 0.05; **p < 0.01; ***p < 0.001; Abbreviations: COM, conditioned medium of human adipose-derived stem cells; PBM, photobiomodulation

All *p* values were related to least significant differences (LSD) test. All treatment groups had lesser colony-forming units (CFU) s of MRSA contrasted to group one (all, *p* < 0.001). h-ASC-COM + PBM, and PBM protocols had lesser CFU than h-ASC-COM (*p* = 0.002, *p* = 0.007), (Fig. 2).

### Wound closure rate (WCR), day 16

**Fig. 3:**
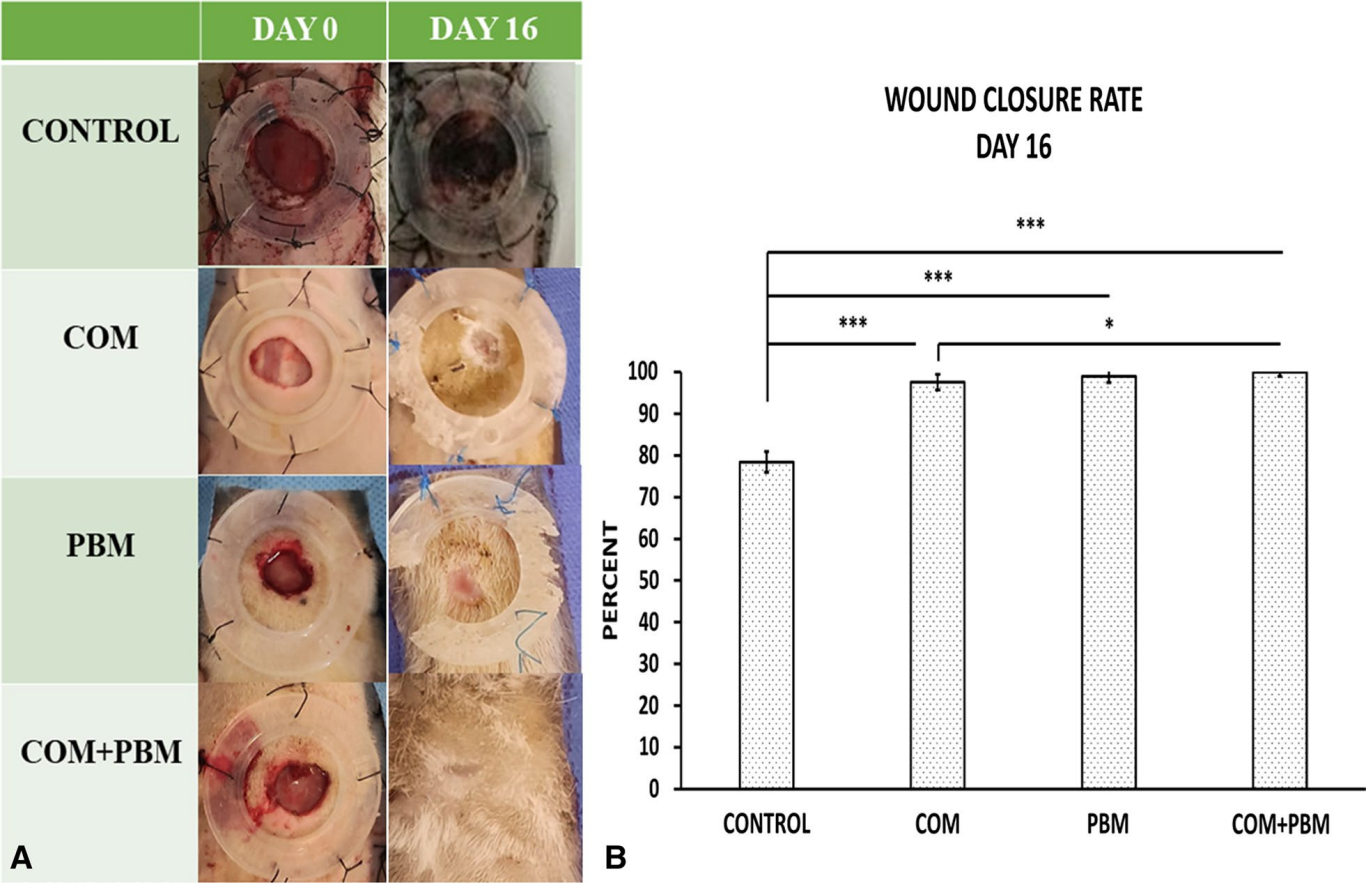
Photos of wounds of all studied groups on days zero, and 16 (**A**).Comparison of wound closure ratio of repairing wounds among studied groups on day 16 (**B**); data reported as mean ± S.D. and ANOVA and LSD tests were applied for analysis. *p < 0.05; ***p < 0.001

There were higher WCR in favor of all experimental groups than group1 (all, *p* < 0.001). WCR of h-ASC-COM + PBM was higher than h-ASC-COM only regime (*p* = 0.025), (Fig. 3).

### Findings of wound strength examination

**Fig. 4:**
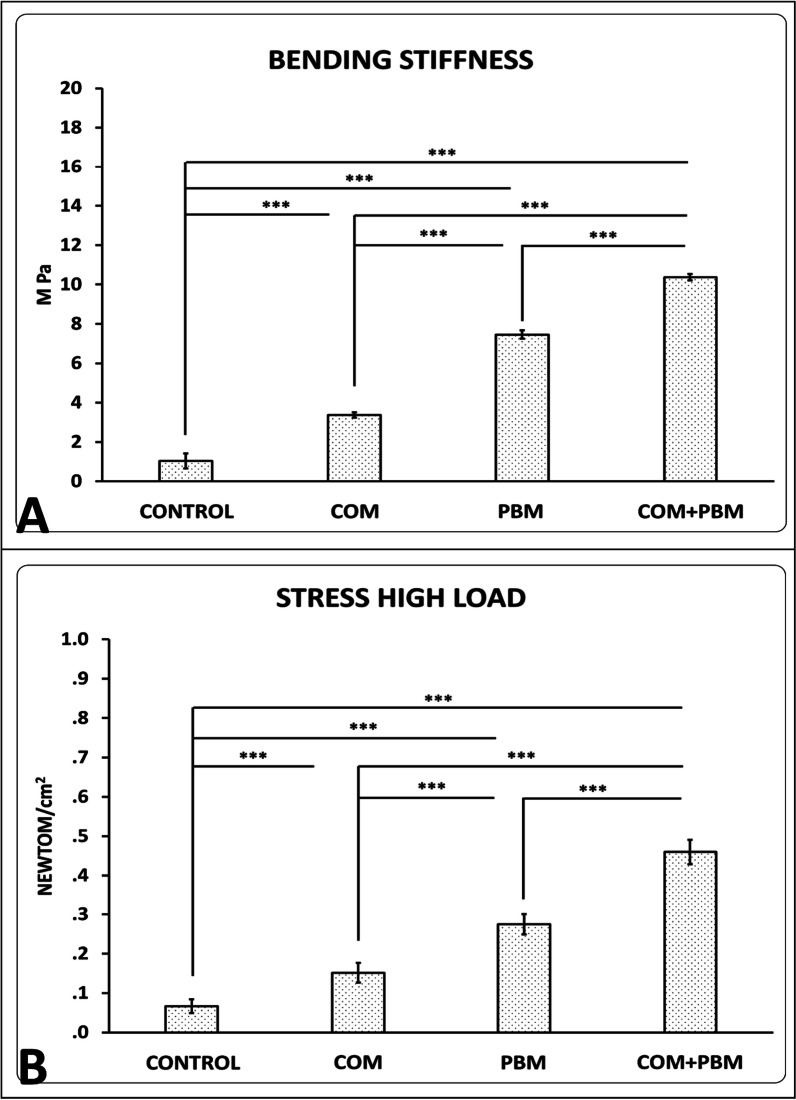
Comparing of bending stiffness (panel** A**), and stress high load (panel** B**) of repairing wounds among studied groups on day 16; data reported as mean ± S.D. and analysis applied with ANOVA and LSD tests. *p < 0.05; **p < 0.01; ***p < 0.001

There were higher bending stiffness (M Pa), (Fig. 4, panel A), and stress high load (N/cm^2^) (Fig. 4, panel B) in favor of all cure groups than group1 (all, *p* < 0.001). These parameters in groups 3, 4 were higher than group 2 (both, *p* < 0.001). Bending stiffness and stress high load of group 4, was higher than group3 (*p* < 0.001).

### Outcomes of stereological examination on day 16, neutrophils, and macrophages, isolated, and together

**Fig. 5:**
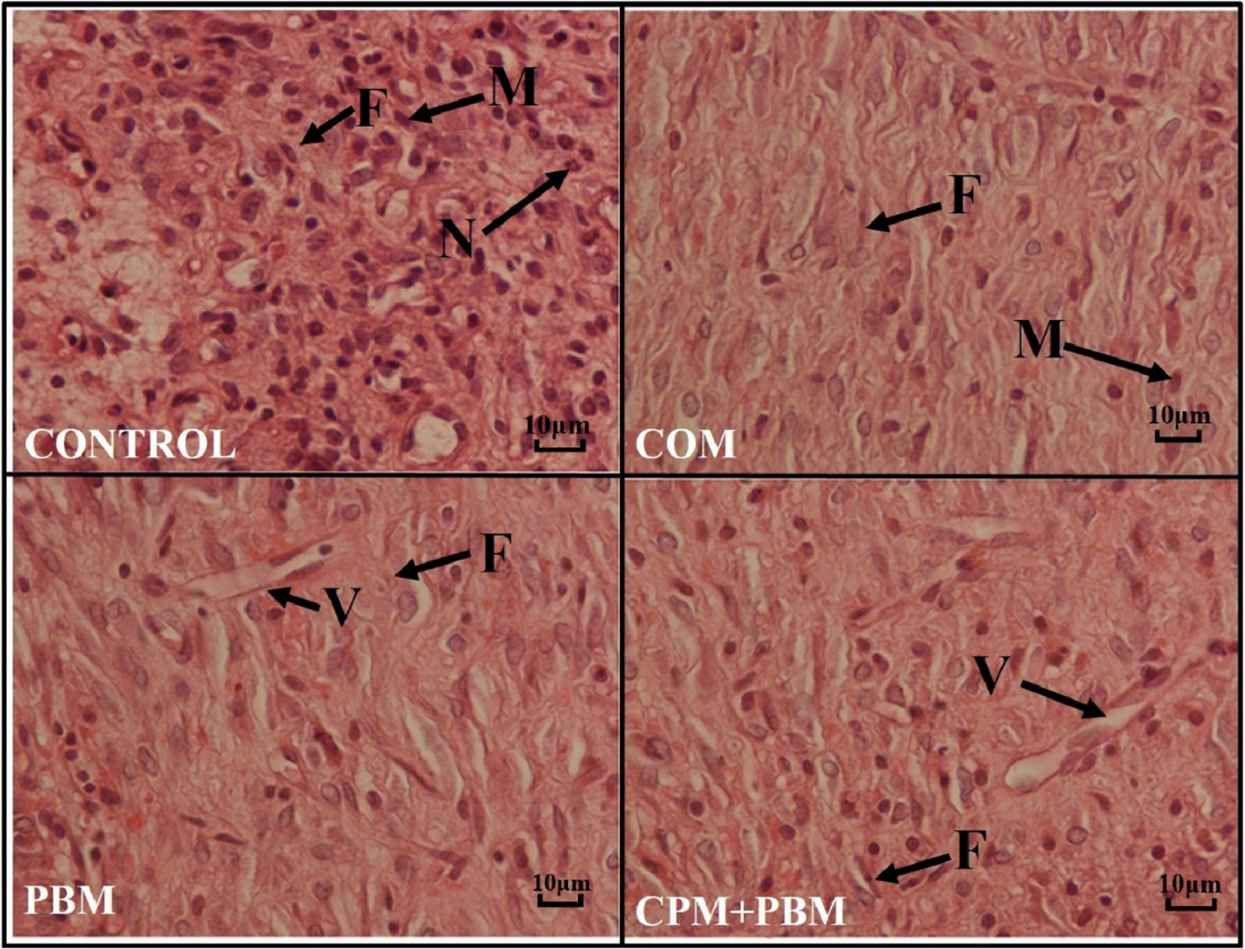
On day 16, Hematoxylin and Eosin staining was considered to get typical histological pictures of the injury sites from the four studied groups. The images depict various cellular components such as fibroblasts (F), macrophages (M), neutrophils (N), and blood vessels (V). The magnification used for capturing these images was 40 × 10

**Fig. 6:**
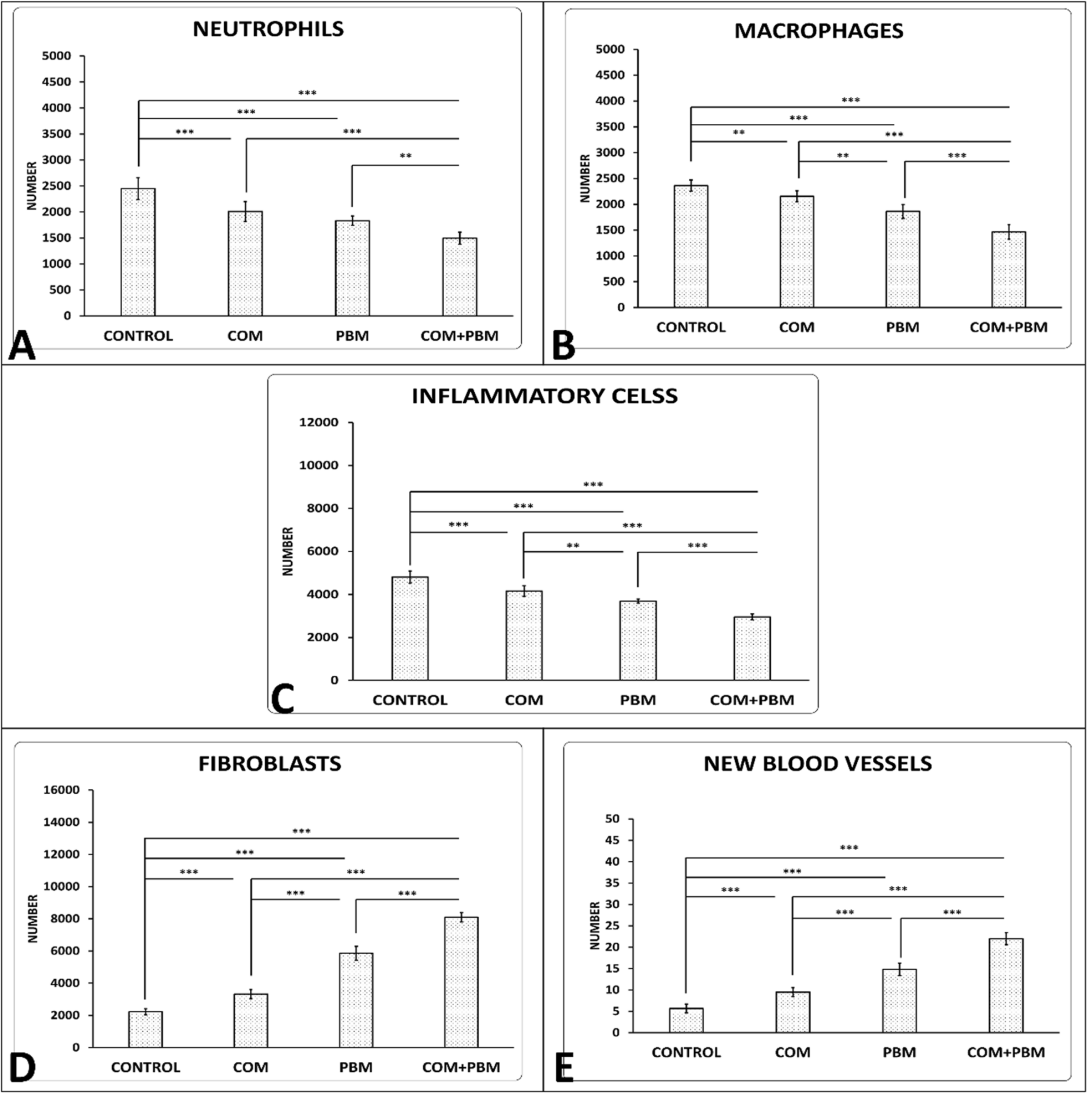
Comparison of the count of neutrophils (panel **A**), macrophages (panel **B**), total inflammatory cells (panel C), fibroblasts (panel **D**), and new blood vessels (panel **E**) of wounds among studied group on day 16; data reported as mean ± S.D. and analysis by using ANOVA and LSD tests; **p < 0.01; ***p < 0.001

On day 16, Fig. 5 displays illustrative histological pictures of the wound regions in the four experimental groups. There were fewer neutrophils (Fig. 6, panel A) and macrophages (Fig. 6, panel B) in favor of all cure regimes than group1 (almost all, *p* < 0.001). Number of these cells were less in group 4 comparing the groups 2, and 3 (almost all, *p* < 0.001). Number of macrophages were less in group 3 in comparison with group 2 (*p* = 0.001).

### Total of number of neutrophils and macrophages

There were fewer total count in numbers of neutrophils and macrophages in favor of all cure regimes than group1 (b, *p* < 0.001). Total count of inflammatory cells were lower in groups 4, 3 than group 2 (both *p* < 0.001, *p* = 0.001). Total count of inflammatory cells were lower in group 4 than group3 (*p* < 0.001) (Fig. 6, panel C).

### Number of fibroblasts, and new blood vessels

There were higher fibroblasts (Fig. 6, panel D), and new blood vessels (Fig. 6, panel E) in favor of all cure regimes than group1 (all, *p* < 0.001). Number of these elements were upper in groups 4, 3 than groups 2, (both, *p* < 0.001). Number of these elements were upper in group 4 than group3 (*p* < 0.001).

All raw data of evaluating methods were shown in Table 1.

**Table 1:** Mean and standard deviation (S. D.) of all row data of entire evaluating methods of studied groups

Groups		Blood sugar, day 0	Blood sugar, day16	Body weight, day 0	Body weight, day16
Control	Mean	370	521	194	156
	S.D.	28	46	26	24
COM	Mean	410	412	191	159
	S.D.	70	69	16	15
PBM	Mean	417	365	200	180
	S.D.	82	73	26	26
COM + PBM	Mean	435	359	224	206
	S.D.	81	76	34	34

Groups		*Staphylococcus aureus,* day16			
Control	Mean	5200			
	S.D.	653			
COM	Mean	878			
	S.D.	84			
PBM	Mean	308			
	S.D.	40			
COM + PBM	Mean	185			
	S.D.	28			

Groups		Wound closure ratio, day, 16			
Control	Mean	78			
	S.D.	2.4			
COM	Mean	97			
	S.D.	1.8			
PBM	Mean	99			
	S.D.	1.5			
COM + PBM	Mean	100			
	S.D.	.1			

Groups		Stress high load		Bending stiffness	
Control	Mean	0.06		1.0	
	S.D.	0.01		0.38	
COM	Mean	0.15		3.37	
	S.D.	0.02		0.13	
PBM	Mean	0.27		7.4	
	S.D.	0.02		0.20	
COM + PBM	Mean	0.46		10.37	
	S.D.	0.03		0.15	

Groups		Neutrophils	Macrophages	Inflammatory cells	Fibroblasts	Blood vessels
Control	Mean	2449	2363	4812	2227	5.66
	S.D.	209	109	287	180	1.03
COM	Mean	2009	2155	4164	3323	9.50
	S.D.	191	105	249	281	1.04
PBM	Mean	1832	1861	3694	5862	14.83
	S.D.	89	135	58	435	1.47
COM + PBM	Mean	1494	1464	2958	8097	22.00
	S.D.	115	141	136	292	1.41

*COM* conditioned medium of human adipose-derived stem cells; *PBM* Photobiomodulation.

## Discussion

The results of all evaluated methods, comprising WCR, microbiological, tensiometrical, and stereological examinations, followed almost identical patterns. While the outcomes of h-ASC-COM + PBM, PBM, and h-ASC-COM regimes were significantly better for all evaluated methods than those of the placebo (all, *p* < 0.001), PBM and h-ASC-COM + PBM treatments achieved superior results than h-ASC-COM only (p = 0.05 to *p* < 0.001). In terms of tensiometrical, and stereological examinations the results of h-ASC-COM + PBM were greater in PBM only (all, *p* < 0.001).

Some recent studies have shown that stem cell therapy did not support diabetic wound restoration. Cianfarani et al. [27] found that diabetic ASCs demonstrated diminished proliferative capacity and migration, releasing lower amounts of important growth factors involved in wound restoration. In addition, the conditioned medium of diabetic ASCs displayed decreased capability to stimulate epidermal cells and fibroblast propagation and immigration. Cianfarani et al. [27] concluded that the conversion process of D. M. converts the intrinsic properties of ASCs, leads to the impairment of their functionality. This, in turn, negatively impacts the therapeutic potential of autologous therapy for DFUs [27]. Dong et al. [28] revealed that normal ASCs and diabetic ASCs could promote wound restoration in rats with simple defect injuries. Still, diabetic ASCs have no significant effect on wound restoration in rats with D.M., which might be attributed to the inhibition of ASCs proliferation and the influence of hyperglycemia and advanced glycation end products (AGE)s intervention on their secretory function [28].

Although MSCs play a critical role in the healing process of skin wounds, investigations have demonstrated that the contribution of transplanted MSCs in repairing wounds within the host tissue is limited due to their low survival rate and lack of proper homing [29]. However other studies have revealed that ASCs have therapeutical impacts on various diseases, and its advantageous effects by paracrine function of the cells [30, 31]. Therefore Fui et al. [32], in their investigation postulated that ASC‐COM is a cell free therapy cause reduction some limitation of MSC therapy. The COMs harmonize the inflammation, increase proliferation and re‐modeling of diabetic wounds [32].

In the line with the Fui et al. [32] conclusion, in current study we found that application of h-ASC-COM on IIDHWM resulted in significantly increases in wound strength, WCR, fibroblasts, and new blood vessels counts than control group. There was also significantly lower inflammatory reaction, and lower CFU of MRSA, compared to control group. In two recent studies other scientists have supported our results. In the first probe, Saheli et al. [33] displayed the beneficial advantages of MSCs-COM on fibroblast performances and wound restoration. Saheli et al. [33] concluded therapy with MSCs-COM could be a novel approach to treat chronic diabetic wound in subjects. In the second probe, Takahashi et al. [34] demonstrated that hydrogels containing MSC -COM accelerated wound restoration in diabetic mice by enhancing angiogenesis, accelerating reepithelialization, and destroying inflammatory reaction. Takahashi et al. [34] concluded topical application of MSC-COM could be a novel treatment for DFUs.

Recently Kim et al. [35] found that mix therapy of spheroidal culture (S.C.) and laser irradiation remarkably improve the angiogenic factor in h-ASCs. Increased migration, viability, and angiogenesis are recorded in cells therapy with COM obtained from the S.C. group. Furthermore, Kim et al. [35] conducted in vivo study using a mice rear limb ischemic simulation; the outcomes revealed that COM derived from cultured spheroids under influence of laser-radiated h-ASCs induced increased angiogenesis in vivo. Kim et al. [35] concluded that, using laser to promote stem cells could solve the major disadvantages of COM-based therapies. On the other hand several investigations studied the effects of BM- MSC-COM applied with PBM for infected wounds therapy [19, 36–39]. According the diabetic wounds, the outcomes were controversial. Three investigations noted that BM-MSC-COM, PBM, and the joint therapy of BM-MSC-COM + PBM improved wound restoration in non-infected diabetic wounds as compared with control group [36–38]. In Pouriran et al. [36] study, while PBM and BM-MSC-COM, alone or combined, boosted biomechanical factors in the wounds, PBM recognized to be more helpful compared to the BM-MSC-COM only. In relation of diabetic wounds that are infected, it was distinguished that both the PBM + BM-MSC-COM and PBM groups were capable to reduce the number of CFUs (colony forming units) and accelerate the process of wound healing than the controls. However, this effect was not reported when BM-MSC-COM alone was applied [19, 39].

In this regard in the current study, in the next step we added PBM, and h-ASC-COM + PBM treatment protocols to our study groups. While the outcomes of h-ASC-COM + PBM, PBM, and h-ASC-COM regimes were significantly better for all evaluated methods than those of the control gathering group (all, *p* < 0.001), PBM and h-ASC-COM + PBM treatments achieved superior results than h-ASC-COM alone (p = 0.05 to *p* < 0.001). Our outcomes were in the same line of those of Fridoni et al. [39] and Kouhkheil et al. [19] investigations.

In terms of tensiometrical, and stereological examinations the results of h-ASC-COM + PBM were superior to those of PBM only (all, *p* < 0.001).

It is vital to distinguish MSC-COM features from those of culture media alone. MSC-COM refers to a type of culture where MSCs are built over a specific time period and in specific conditions. On the other hand, culture media is clearly the substance applied to cultivate MSCs. In some investigations, culture media was operated as the control group to demonstrate the favorable effects of MSC-COM in the healing of wounds [40–42]. Higher as well as faster wound restoration rates were observed with MSC-COM treatment than culture medium itself. The study distinguished that using MSC-COM topically after laser therapy resulted in lower level of skin redness and darkening in comparison with using the culture medium alone [43]. These data suggest that the effects observed of MSC-COM in these diseases is due to the conditioned medium and is not being caused by the culture medium alone.

One drawback in usage of MSC-COM is that in order to apply it on patients, the exact composition of each conditioned medium must be identified. Moreover, conditioned medium usually requires to be administered more frequently than MSCs because the cytokines and growth factors found in the medium have shorter half-lives in comparison with the stem cells, which may survive for longer periods of time [44]. Further studies are needed to identify the specific constituents of MSC-COM that effectively treat cutaneous disorders. Additionally, it is essential to establish regulatory guidelines for the production, standardization, and quality control of MSC-COM with regard to the source of the MSCs, methods of isolation, and culture conditions. Including measures would be necessary to ensure the safety and efficacy of these products [45].

In the current study, we could not characterize either the exact composition of the condition medium or critical signaling molecules that may potentially be secreted by the h-ASC in the h-ASC-COM, which could be considered as two important limitations. On the other hand, some research has indicated that a mixture called h-ASC-COM, made up of amino acids, vitamins, glucose, and human serum, can generate therapeutically significant conditioned medium (COM) with angiogenic and/or antiapoptotic properties. This CM including factors such as vascular endothelial cell growth factor, fibroblast growth factor 2, hepatocyte growth factor, and chemokine (C-X-C motif) ligand 12 [46].

We should note that we purchased 24 adult male Wistar rats from the Pasteur Institute of Iran, all aged 3 months. These rats were then randomly divided into four groups, each containing six animals. In each rat, both a TIIDM and a wound for delayed healing (IIDHWM) were created. These rats received ethical treatment in a standardized animal facility, with consistent care provided regardless of their group assignments. The initial differences in body weights and blood glucose levels observed among the study groups could be attributed to individual responses of rats to TIIDM and IIDHWM. Consequently, for the allocation of animals to the study groups, careful attention was given to the pivotal role of randomization in experimental research. It is important to note that the statistical tests revealed no significant differences in the initial body weights and blood glucose levels among the groups under study.

Interestingly, after 17 days of treatments, several noteworthy changes were observed that warrant further attention. As the rats all experienced relatively large delayed healing ischemic wounds, a decline in body weights could be reasonably expected. Notably, while treatment with COM prevented further escalation in blood glucose levels on day 16 compared to day zero, treatments involving PBM (groups 3 and 4) lowered blood glucose to levels below those of day zero. Some prior studies align with our findings:

Several studies have demonstrated the favorable effects of PBM on glucose metabolism in hyperglycemic and diabetic individuals. Sene-Fiorese et al. conducted research on obese females, finding that five months of PBM combined with physical activity led to a more substantial decrease in blood insulin levels compared to physical activity combined with placebo PBM [47]. Francisco et al. reported that PBM using light-emitting diodes, along with mild exercise, significantly lowered blood glucose values in TIIDM patients [48]. Silva et al. [49] noted that daily PBM over one month reduced blood glucose in obese mice. These studies’ outcomes support our discovery of reduced blood glucose levels following PBM treatment in TIDM rats. However, the precise mechanism by which PBM impacts blood glucose metabolism remains incompletely understood. To the best of our knowledge, the positive effects of PBM on blood glucose levels during the healing process of diabetic skin wounds have not been previously reported. Thus, our current research is the first to demonstrate that PBM treatment of diabetic wounds over 15 days leads to significant decreases in blood glucose levels. PBM seems to enhance mitochondrial metabolism by increasing CCO oxidase activity, thereby promoting increased glucose consumption. Correspondingly, a previous study found that PBM elevated citrate synthase activity and Krebs cycle function. The heightened glucose consumption for ATP production could explain the blood glucose reduction [1, 50]. PBM might also lower blood glucose levels by inhibiting the c-Jun N-terminal kinase pathway and promoting protein kinase B phosphorylation [49]. Nevertheless, further studies are warranted in this area.

In the COM group, we observed a greater number of new blood vessels and a reduced inflammatory reaction compared to the control group. Our findings are consistent with the studies by Iron et al. [51] and Zhang et al. [52]. In their original article from 2018, Iron et al. [51] concluded that topical application of COM accelerated diabetic wound healing in a swine model, with enhanced angiogenesis and immunomodulation playing significant roles. In their review article, Zhang et al. [52] stated that the use of COM yielded positive effects on wound healing both in vitro and in vivo.

In the PBM group of our study, we observed more fibroblasts, new blood vessels, increased volumes of new epidermis and dermis, and a decreased inflammatory reaction compared to the control group. Our results are in line with Houreld’s research. In her review article from 2019, Houreld concluded that PBM, which involves the non-invasive application of light at specific wavelengths, has been shown to expedite the healing of chronic wounds, including diabetic foot ulcers (DFUs). PBM induces photophysical and photochemical changes within cells without causing thermal damage. It has been demonstrated to promote tissue regeneration and hasten wound repair by mitigating inflammation and oxidative stress, accelerating cell migration and proliferation, and fostering extracellular matrix production as well as the release of crucial growth factors [53].

## Conclusions

PBM, h-ASC-COM, and h-ASC-COM + PBM treatments meaningfully accelerated the maturation phase of the wound restoration process in IIDWHM with MRSA in TIIDM rats by diminishing the inflammatory reaction, and the CFU of MRSA; and increasing wound strength, WCR, number of fibroblasts, and new blood vessels. While the h-ASC-COM + PBM, and PBM were more suitable than the effect of h-ASC-COM, the results of h-ASC-COM + PBM were superior to PBM alone.

### Our recommendations for more research

Further experimental probes are required to understand in greater details the molecular mechanisms of these modalities, particularly the h-ASC-COM + PBM management protocol in diabetic infected wound restoration. It is also suggested that clinical trials be conducted in this field.

## Methods

### Animals and groups

**Fig. 7:**
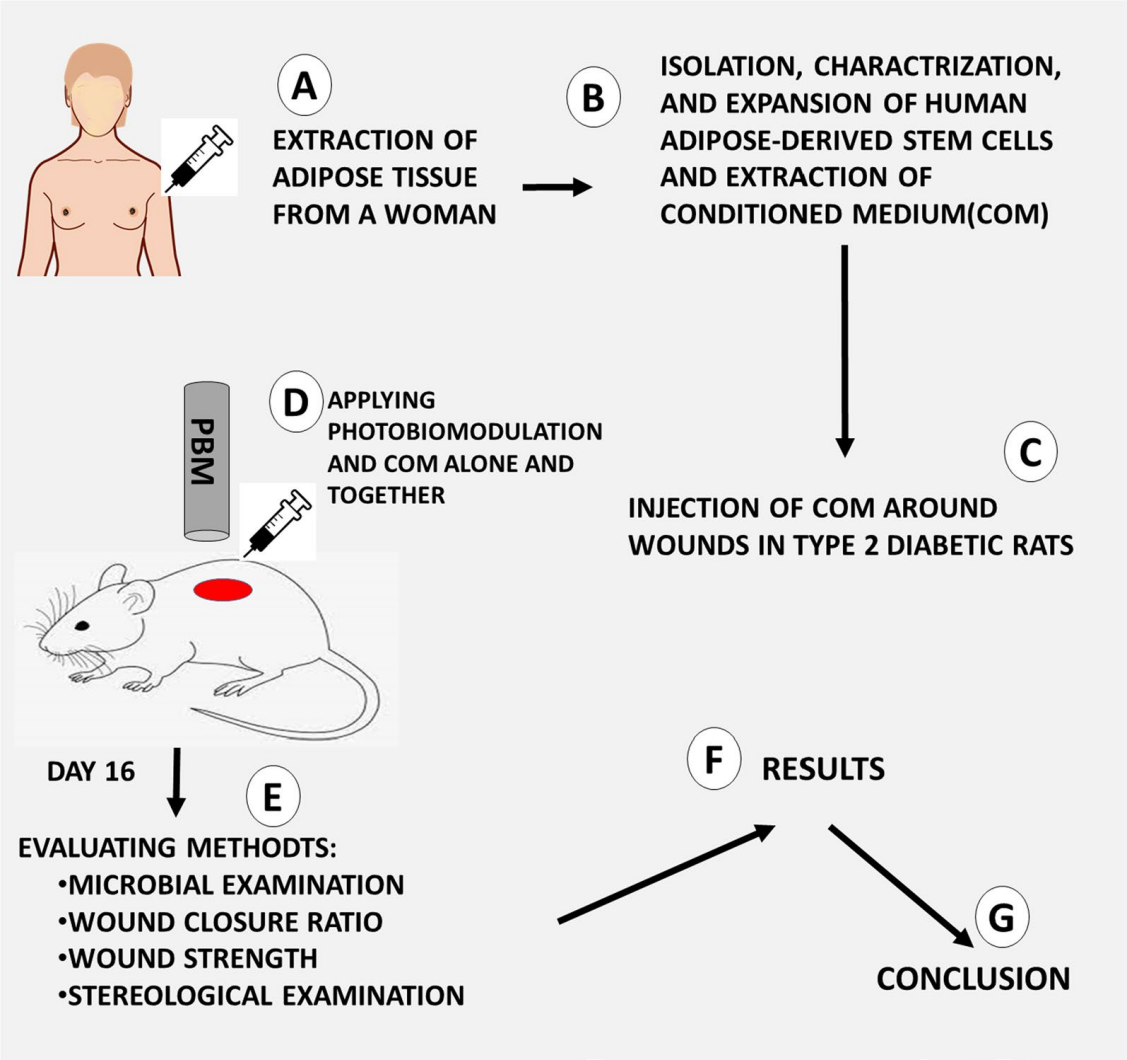
Applying photobiomodulation and condition medium alone and together

h-ASCs were donated, their immunophenotypic characteristics were analyzed, and their COM was prepared by concentrating h-ASCs. We bought 24 Wistar male adult rats with age of 3 month old from Pasteur Institute of Iran. They were randomly divided into four groups, six animals each group. TIIDM, and a delayed healing wound (IIDHWM) was made in each rats. They were ethically treated in a standard animal home and were received similar cares regardless of their groups. The first group was considered the control group (placebo); the second group received h-ASC-COM, the third group; was exposed to PBM; and group four received h-ASC-COM + PBM. On day 16, clinical and laboratory examinations were done. Rats were euthanized with CO_2_ on day 16, and 2 study samples from each wound for tensiometerical and stereological examinations were extracted. There were six samples in each group for all evaluating methods. We obtained approval from the Ethics Board (IRB) at Shahid Beheshti University of Medical Sciences (SBMU.MSP.REC.1400.456). A diagram of study design was added, (Fig. 7).

### Isolation and expansion of h-ASCs

Subdermal fat was donated by a healthy adult lady who is undertaking plastic surgery with a knowledgeable patient agreement. Approximately five cubic centimeters of adipose tissue were extracted, then subjected to standard methods of cultivation and proliferation. The resulting cells were assessed for MSC markers using flow cytometry following the previously described protocol. The h-ASCs attained from passage 4 were detached and further used [54].

### Preparation of h-ASC-COM

h-ASC-COM was prepared by cultivating 10^6^ h-ASC s from passage 4. According to the manufacturer’s protocol, COM was gathered and condensed roughly tenfold via lyophilized-drying (Christ Alpha1-2 LD Plus, Germany) [36].

### Induction of TIIDM

At first, the animals were given a drink containing 10% fructose (Biobasic, Canada) rather the regular water, and they were fed a standard pellet of rats for 14 days. In the next step, streptozotocin [(STZ), 40 mg/kg Santa Cruz Biotechnology, Inc., Dallas, TX, USA] was injected. Seven days later, the rat’s blood sugar level were checked. Rats with high blood sugar levels (more than 250 mg/dl) for 21 days after injection of STZ were considered as TIIDM [20]. An oral glucose tolerance test was done in our previous study [20].

### Operation for wounding

**Fig. 8:**
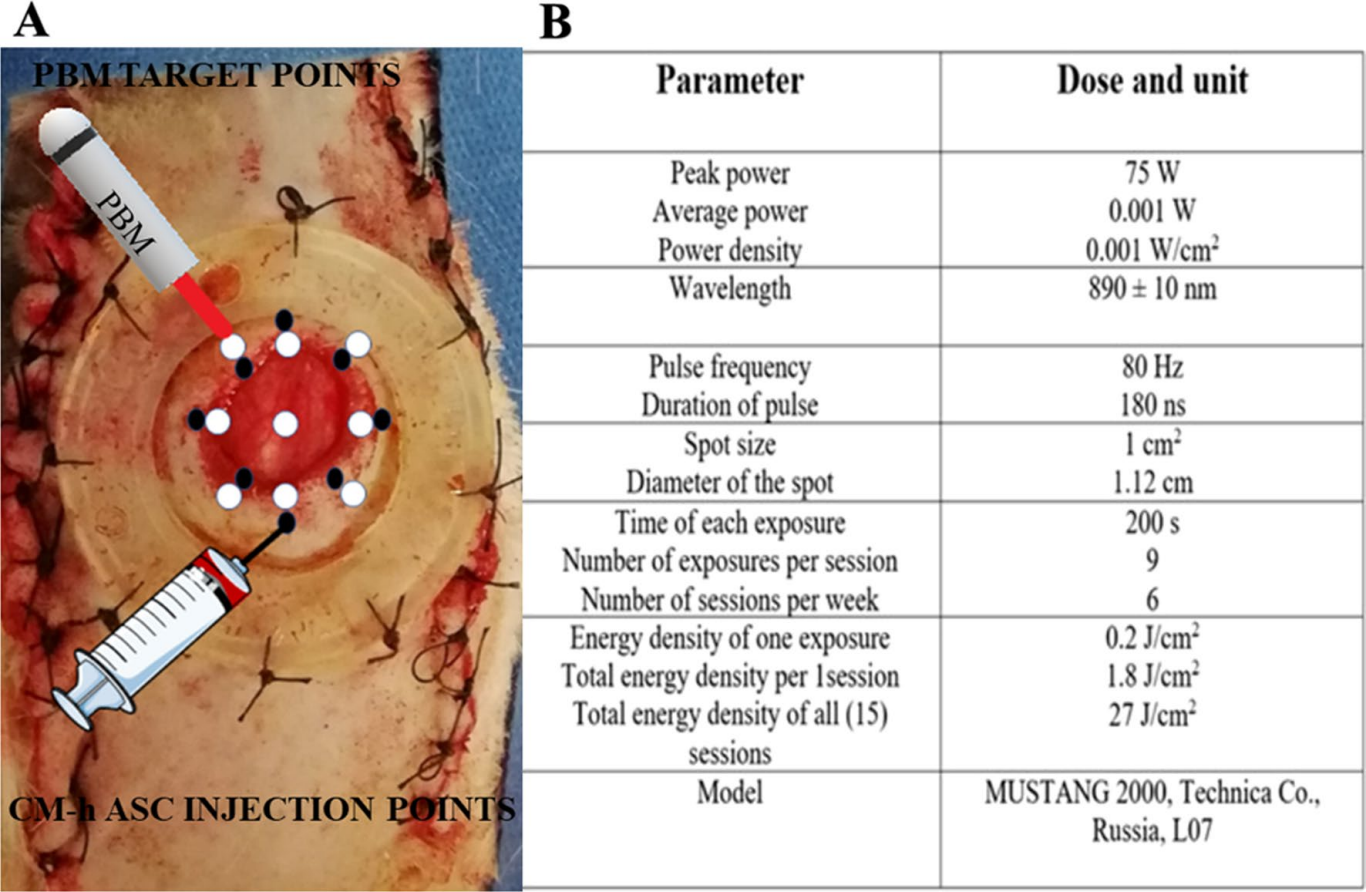
Wound area, silicon ring around the edge of wound, condition medium injection points, PBM irradiation points (Panel **A**), and the PBM specifications (Panel **B**)

Under total anesthesia and sterile circumstances, two parallel 10-cm incisions were induced in each animal and were sutured. The distance between them was 3.5 cm. Next, a 12-mm, all-layer, round skin injury was produced in the middle area between two incisions. Finally, a ring-shaped skin holder was properly sutured around the excision to avoid the panniculus carnosus muscle contraction (Fig. 8A) [18].

### Inoculation of infection into the wounds

Patients who suffer from DFUs may have methicillin-resistant *Staphylococcus aureus* (MRSA) occurs in 10–32% of their infections. MRSA is heavily connected to a higher chance of treatment not working, increased illness, and higher hospitalization expenses for these patients [55]. Therefore, in the current experiment, a standard MRSA strain (ATCC 25923, Seattle 1945, USA) was used as described in a prior study. A 100-μl aliquot of the colony with 2 × 10^7^ MRSA was administered locally in each injured area soon after wound formation. The number of each bacteria per probable specie was counted as CFUs. On day 16, the CFU of wounds in all groups was counted [18].

### Gross examinations

Throughout the experiment, the rats' weights and blood sugar levels were checked.

### h-ASC-COM and PBM administrations

300 λ of the PBS containing tenfold concentrated COM of 10^6^ h-ASC were administered (i. p.) on days 1,3,5, and 7, to 8 points around each wound of rats of groups 2 and 4 (Fig. 6A) [39]. The wounds in groups 3, and 4 were radiated with a PBM generator, and complete information on PBM was provided in Fig. 8B [18].

### Measurement of WCR

On day 16, all wounds were photographed using standard methods, and the wound area was measured by utilizing IMAGE J software developed by the National Institutes of Health (NIH). Subsequently, WCRs were calculated based on this data [18].

$$ {\text{WCR}} = \left[ {\left( {{\text{wound surface on day }}0 - {\text{open wound surface on day X}}} \right)/{\text{wound surface on day }}0} \right] \times {1}00\% $$

### Wound strength examination

One 0.5 × 5 cm sample of repairing area was scaled on a material testing machine (Santam, Iran). The deformation rate was counted at 0.166 mm/s. From the stress–strain graph, bending stiffness (MPa) and high-stress load (N/cm^2^) were calculated [18].

### Stereological procedure by physical dissector method

10% Formaldehyde -fixed samples were embedded in paraffinic blocks, and ten sections of 5-μm thickness were sequentially sliced and stained with the H&E method. Numerical density (Nv) and total (N) counts of fibroblasts, macrophages, and neutrophils in 6 fields of wound area were examined by a light microscope at a magnification of 40 × 10 [56].

### Count of cells

$$ {\text{Nv}} = \Sigma {\text{Q}}/({\text{h}} \times {\text{a}}/{\text{f}} \times \Sigma {\text{P}}) $$

ΣQ = number of cells; h = dissector height; a/f = area of all counting frames; and ΣP = the total number of test points hitting the structure [56].

$$ {\text{N}} = {\text{Nv}} \times {\text{V }}({\text{volume}}) $$

$$ {\text{Number of new blood vessels}} = {2}\Sigma {\text{Q}}/\left( {\Sigma {\text{P}} \times {\text{a}}/{\text{f}}} \right) $$

ΣQ = total number of vessels per wound. Number of new blood vessels was measured as an angiogenesis marker.

### Statistical analysis

Our study reported data in the format of mean ± standard deviation (S.D.). To determine any considerable differences between the two groups, the LSD test was used for values that had a normal distribution. At the same time, one way analysis of variance (ANOVA) was applied to compare data among multiple groups. A *p* value lesser than 0.05 was considered significant. The statistical analysis of Tensiometry, Stereology, Wound Closure Rate, Blood Sugar, Body Weights, and Microbe Analysis in this study is available as an Additional file 1.

### Supplementary Information

**Additional file 1.** The statistical analysis of Tensiometry, Stereology, Wound Closure Rate, Blood Sugar, Body Weights, and Microbe Analysis.

## Data Availability

The statistical outcomes are available as an Additional file 1 no one.

## Abbreviations

ANOVA: Analysis of variance
BM-MSC-COM: Bone-marrow-derived mesenchymal stem cell‐conditioned medium
CD: Cluster of differentiation
CFU: Colony-forming unit
D.M.: Diabetes mellitus
DFD: Diabetic foot disease
DFU: Diabetic foot ulcers
DPN: Diabetic polyneuropathy
h-ASC-COM: Conditioned medium of human adipose-derived stem cells
h-ASCs: Human adipose-derived stem cells
IIDHWM: Ischemic infected delayed-healing wound model
LSD: Least significant differences
MRSA: Methicillin-resistant *Staphylococcus aureus*
PBM: Photobiomodulation
S.D.: Standard deviation
STZ: Streptozotocin
TI DM: Type 1 diabetes mellitus
TII DM: Type 2 diabetes mellitus
WCR: Wound closure ratio
