# Supplementary material for: Promising improvement in infected Wound Healing in Type two Diabetic rats by Combined effects of conditioned medium of human adipose-derived stem cells plus Photobiomodulation
Source: Lab Anim Res. 2023 Nov 15;39:29. doi: 10.1186/s42826-023-00178-z (PMC10648630; doi:10.1186/s42826-023-00178-z)
Supplement: Supplementary file 1 — Additional file 1. The statistical analysis of Tensiometry, Stereology, Wound Closure Rate, Blood Sugar, Body Weights, and Microbe Analysis. [file 42826_2023_178_MOESM1_ESM.docx]

Tensiometry

| **Case Summaries** | | | | | |
| --- | --- | --- | --- | --- | --- |
| Groups | | Force | Stress | Energy | Bending |
| Control | Mean | .7217 | .0667 | .8600 | 1.0333 |
|  | Std. Deviation | .16030 | .01751 | .26840 | .38795 |
| CM | Mean | 1.5850 | .1517 | 2.7317 | 3.3700 |
|  | Std. Deviation | .23210 | .02483 | .13014 | .13476 |
| PBM | Mean | 2.8067 | .2750 | 5.8583 | 7.4550 |
|  | Std. Deviation | .25766 | .02588 | .92183 | .20936 |
| CM+PBM | Mean | 4.6567 | .4600 | 9.3967 | 10.3700 |
|  | Std. Deviation | .31551 | .03098 | .35012 | .15799 |
| Total | Mean | 2.4425 | .2383 | 4.7117 | 5.5571 |
|  | Std. Deviation | 1.52683 | .15288 | 3.34551 | 3.69059 |

| **ANOVA** | | | | | | |
| --- | --- | --- | --- | --- | --- | --- |
|  | | Sum of Squares | df | Mean Square | F | Sig. |
| Force | Between Groups | 52.390 | 3 | 17.463 | 284.537 | .000 |
|  | Within Groups | 1.228 | 20 | .061 |  |  |
|  | Total | 53.618 | 23 |  |  |  |
| Stress | Between Groups | .525 | 3 | .175 | 274.030 | .000 |
|  | Within Groups | .013 | 20 | .001 |  |  |
|  | Total | .538 | 23 |  |  |  |
| Energy | Between Groups | 252.119 | 3 | 84.040 | 316.730 | .000 |
|  | Within Groups | 5.307 | 20 | .265 |  |  |
|  | Total | 257.426 | 23 |  |  |  |
| Bending | Between Groups | 312.083 | 3 | 104.028 | 1752.367 | .000 |
|  | Within Groups | 1.187 | 20 | .059 |  |  |
|  | Total | 313.271 | 23 |  |  |  |

**Post Hoc Tests**

| Dependent Variable | (I) Groups | (J) Groups | Sig. |
| --- | --- | --- | --- |
|  |  |  |  |
| Force | Control | CM | .000 |
|  |  | PBM | .000 |
|  |  | CM+PBM | .000 |
|  | CM | Control | .000 |
|  |  | PBM | .000 |
|  |  | CM+PBM | .000 |
|  | PBM | Control | .000 |
|  |  | CM | .000 |
|  |  | CM+PBM | .000 |
|  | CM+PBM | Control | .000 |
|  |  | CM | .000 |
|  |  | PBM | .000 |
| Stress | Control | CM | .000 |
|  |  | PBM | .000 |
|  |  | CM+PBM | .000 |
|  | CM | Control | .000 |
|  |  | PBM | .000 |
|  |  | CM+PBM | .000 |
|  | PBM | Control | .000 |
|  |  | CM | .000 |
|  |  | CM+PBM | .000 |
|  | CM+PBM | Control | .000 |
|  |  | CM | .000 |
|  |  | PBM | .000 |
| Energy | Control | CM | .000 |
|  |  | PBM | .000 |
|  |  | CM+PBM | .000 |
|  | CM | Control | .000 |
|  |  | PBM | .000 |
|  |  | CM+PBM | .000 |
|  | PBM | Control | .000 |
|  |  | CM | .000 |
|  |  | CM+PBM | .000 |
|  | CM+PBM | Control | .000 |
|  |  | CM | .000 |
|  |  | PBM | .000 |
| Bending | Control | CM | .000 |
|  |  | PBM | .000 |
|  |  | CM+PBM | .000 |
|  | CM | Control | .000 |
|  |  | PBM | .000 |
|  |  | CM+PBM | .000 |
|  | PBM | Control | .000 |
|  |  | CM | .000 |
|  |  | CM+PBM | .000 |
|  | CM+PBM | Control | .000 |
|  |  | CM | .000 |
|  |  | PBM | .000 |

Stereology

| **Case Summaries** | | | | | | |
| --- | --- | --- | --- | --- | --- | --- |
| Groups | | Neutrophils | Macrophage | Inflammatory.Cells | Fibroblasts | Vessels |
| Control | Mean | 2449.5556 | 2363.1111 | 4812.6667 | 2227.7778 | 5.6667 |
|  | Std. Deviation | 209.80485 | 109.89018 | 287.17629 | 180.75283 | 1.03280 |
| CM | Mean | 2009.5556 | 2155.3333 | 4164.8889 | 3323.1111 | 9.5000 |
|  | Std. Deviation | 191.42955 | 105.27678 | 249.37777 | 281.68467 | 1.04881 |
| PBM | Mean | 1832.4444 | 1861.5556 | 3694.0000 | 5862.0000 | 14.8333 |
|  | Std. Deviation | 89.47716 | 135.84414 | 58.20271 | 435.21417 | 1.47196 |
| CM+PBM | Mean | 1494.8889 | 1464.0000 | 2958.8889 | 8097.3333 | 22.0000 |
|  | Std. Deviation | 115.32961 | 141.21756 | 136.92864 | 292.60030 | 1.41421 |
| Total | Mean | 1946.6111 | 1961.0000 | 3907.6111 | 4877.5556 | 13.0000 |
|  | Std. Deviation | 381.86412 | 363.91096 | 716.88057 | 2345.92652 | 6.37250 |

| **ANOVA** | | | | | | |
| --- | --- | --- | --- | --- | --- | --- |
|  | | Sum of Squares | df | Mean Square | F | Sig. |
| Neutrophils | Between Groups | 2844012.667 | 3 | 948004.222 | 37.187 | .000 |
|  | Within Groups | 509852.148 | 20 | 25492.607 |  |  |
|  | Total | 3353864.815 | 23 |  |  |  |
| Macrophage | Between Groups | 2738141.926 | 3 | 912713.975 | 59.310 | .000 |
|  | Within Groups | 307775.407 | 20 | 15388.770 |  |  |
|  | Total | 3045917.333 | 23 |  |  |  |
| Inflammatory.Cells | Between Groups | 10986125.852 | 3 | 3662041.951 | 87.821 | .000 |
|  | Within Groups | 833982.519 | 20 | 41699.126 |  |  |
|  | Total | 11820108.370 | 23 |  |  |  |
| Fibroblasts | Between Groups | 124642317.630 | 3 | 41547439.210 | 429.382 | .000 |
|  | Within Groups | 1935220.741 | 20 | 96761.037 |  |  |
|  | Total | 126577538.370 | 23 |  |  |  |
| Vessels | Between Groups | 902.333 | 3 | 300.778 | 189.965 | .000 |
|  | Within Groups | 31.667 | 20 | 1.583 |  |  |
|  | Total | 934.000 | 23 |  |  |  |

**Post Hoc Tests**

| Dependent Variable | (I) Groups | (J) Groups | Sig. |
| --- | --- | --- | --- |
|  |  |  |  |
| Neutrophils | Control | CM | .000 |
|  |  | PBM | .000 |
|  |  | CM+PBM | .000 |
|  | CM | Control | .000 |
|  |  | PBM | .069 |
|  |  | CM+PBM | .000 |
|  | PBM | Control | .000 |
|  |  | CM | .069 |
|  |  | CM+PBM | .002 |
|  | CM+PBM | Control | .000 |
|  |  | CM | .000 |
|  |  | PBM | .002 |
| Macrophage | Control | CM | .009 |
|  |  | PBM | .000 |
|  |  | CM+PBM | .000 |
|  | CM | Control | .009 |
|  |  | PBM | .001 |
|  |  | CM+PBM | .000 |
|  | PBM | Control | .000 |
|  |  | CM | .001 |
|  |  | CM+PBM | .000 |
|  | CM+PBM | Control | .000 |
|  |  | CM | .000 |
|  |  | PBM | .000 |
| Inflammatory.Cells | Control | CM | .000 |
|  |  | PBM | .000 |
|  |  | CM+PBM | .000 |
|  | CM | Control | .000 |
|  |  | PBM | .001 |
|  |  | CM+PBM | .000 |
|  | PBM | Control | .000 |
|  |  | CM | .001 |
|  |  | CM+PBM | .000 |
|  | CM+PBM | Control | .000 |
|  |  | CM | .000 |
|  |  | PBM | .000 |
| Fibroblasts | Control | CM | .000 |
|  |  | PBM | .000 |
|  |  | CM+PBM | .000 |
|  | CM | Control | .000 |
|  |  | PBM | .000 |
|  |  | CM+PBM | .000 |
|  | PBM | Control | .000 |
|  |  | CM | .000 |
|  |  | CM+PBM | .000 |
|  | CM+PBM | Control | .000 |
|  |  | CM | .000 |
|  |  | PBM | .000 |
| Vessels | Control | CM | .000 |
|  |  | PBM | .000 |
|  |  | CM+PBM | .000 |
|  | CM | Control | .000 |
|  |  | PBM | .000 |
|  |  | CM+PBM | .000 |
|  | PBM | Control | .000 |
|  |  | CM | .000 |
|  |  | CM+PBM | .000 |
|  | CM+PBM | Control | .000 |
|  |  | CM | .000 |
|  |  | PBM | .000 |

Wound Closure Rate

| **Case Summaries** | | | | |
| --- | --- | --- | --- | --- |
| Groups | | Wound.Closure.Area.4 | Wound.Closure.Area.8 | Wound.Closure.Area.16 |
| Control | Mean | 8.3703 | 30.6231 | 78.4135 |
|  | Std. Deviation | 2.39082 | 3.29861 | 2.46949 |
| CM | Mean | 22.8889 | 52.2534 | 97.5753 |
|  | Std. Deviation | 2.93477 | 9.80291 | 1.87966 |
| PBM | Mean | 51.8391 | 67.6969 | 99.0131 |
|  | Std. Deviation | 4.01134 | 9.47082 | 1.53124 |
| CM+PBM | Mean | 54.2474 | 72.2184 | 100.0000 |
|  | Std. Deviation | 2.02598 | 1.55787 | .00000 |
| Total | Mean | 34.3364 | 55.6979 | 93.7505 |
|  | Std. Deviation | 20.02288 | 17.86495 | 9.23018 |

| **ANOVA** | | | | | | |
| --- | --- | --- | --- | --- | --- | --- |
|  | | Sum of Squares | df | Mean Square | F | Sig. |
| Wound.Closure.Area.4 | Between Groups | 9048.441 | 3 | 3016.147 | 349.452 | .000 |
|  | Within Groups | 172.622 | 20 | 8.631 |  |  |
|  | Total | 9221.063 | 23 |  |  |  |
| Wound.Closure.Area.8 | Between Groups | 6345.089 | 3 | 2115.030 | 42.492 | .000 |
|  | Within Groups | 995.506 | 20 | 49.775 |  |  |
|  | Total | 7340.595 | 23 |  |  |  |
| Wound.Closure.Area.16 | Between Groups | 1899.630 | 3 | 633.210 | 211.489 | .000 |
|  | Within Groups | 59.881 | 20 | 2.994 |  |  |
|  | Total | 1959.511 | 23 |  |  |  |

**Post Hoc Tests**

| Dependent Variable | (I) Groups | (J) Groups | Sig. |
| --- | --- | --- | --- |
|  |  |  |  |
| Wound.Closure.Area.4 | Control | CM | .000 |
|  |  | PBM | .000 |
|  |  | CM+PBM | .000 |
|  | CM | Control | .000 |
|  |  | PBM | .000 |
|  |  | CM+PBM | .000 |
|  | PBM | Control | .000 |
|  |  | CM | .000 |
|  |  | CM+PBM | .171 |
|  | CM+PBM | Control | .000 |
|  |  | CM | .000 |
|  |  | PBM | .171 |
| Wound.Closure.Area.8 | Control | CM | .000 |
|  |  | PBM | .000 |
|  |  | CM+PBM | .000 |
|  | CM | Control | .000 |
|  |  | PBM | .001 |
|  |  | CM+PBM | .000 |
|  | PBM | Control | .000 |
|  |  | CM | .001 |
|  |  | CM+PBM | .280 |
|  | CM+PBM | Control | .000 |
|  |  | CM | .000 |
|  |  | PBM | .280 |
| Wound.Closure.Area.16 | Control | CM | .000 |
|  |  | PBM | .000 |
|  |  | CM+PBM | .000 |
|  | CM | Control | .000 |
|  |  | PBM | .166 |
|  |  | CM+PBM | .025 |
|  | PBM | Control | .000 |
|  |  | CM | .166 |
|  |  | CM+PBM | .335 |
|  | CM+PBM | Control | .000 |
|  |  | CM | .025 |
|  |  | PBM | .335 |

Blood Sugar+Weights

| **Case Summaries** | | | | | |
| --- | --- | --- | --- | --- | --- |
| Groups | | BS.Day0 | BS.Day16 | Wei.Day0 | Wei.Day16 |
| Control | Mean | 370.0000 | 521.1667 | 194.8333 | 156.6667 |
|  | Std. Deviation | 28.39718 | 46.50771 | 26.23293 | 24.36117 |
| CM | Mean | 410.8333 | 412.1667 | 191.6667 | 159.0000 |
|  | Std. Deviation | 70.21515 | 69.52530 | 16.46410 | 15.94992 |
| PBM | Mean | 417.1667 | 365.6667 | 200.6667 | 180.5000 |
|  | Std. Deviation | 82.84061 | 73.29302 | 26.91963 | 26.99444 |
| CM+PBM | Mean | 435.0000 | 359.8333 | 224.0000 | 206.1667 |
|  | Std. Deviation | 81.81687 | 76.98939 | 34.84250 | 34.85063 |
| Total | Mean | 408.2500 | 414.7083 | 202.7917 | 175.5833 |
|  | Std. Deviation | 69.17354 | 91.37047 | 28.23655 | 31.97134 |

| **ANOVA** | | | | | | |
| --- | --- | --- | --- | --- | --- | --- |
|  | | Sum of Squares | df | Mean Square | F | Sig. |
| BS.Day0 | Between Groups | 13588.833 | 3 | 4529.611 | .939 | .440 |
|  | Within Groups | 96465.667 | 20 | 4823.283 |  |  |
|  | Total | 110054.500 | 23 |  |  |  |
| BS.Day16 | Between Groups | 100537.125 | 3 | 33512.375 | 7.327 | .002 |
|  | Within Groups | 91479.833 | 20 | 4573.992 |  |  |
|  | Total | 192016.958 | 23 |  |  |  |
| Wei.Day0 | Between Groups | 3848.458 | 3 | 1282.819 | 1.771 | .185 |
|  | Within Groups | 14489.500 | 20 | 724.475 |  |  |
|  | Total | 18337.958 | 23 |  |  |  |
| Wei.Day16 | Between Groups | 9554.167 | 3 | 3184.722 | 4.564 | .014 |
|  | Within Groups | 13955.667 | 20 | 697.783 |  |  |
|  | Total | 23509.833 | 23 |  |  |  |

**Post Hoc Tests**

| Dependent Variable | (I) Groups | (J) Groups | Sig. |
| --- | --- | --- | --- |
|  |  |  |  |
| BS.Day0 | Control | CM | .321 |
|  |  | PBM | .253 |
|  |  | CM+PBM | .121 |
|  | CM | Control | .321 |
|  |  | PBM | .876 |
|  |  | CM+PBM | .553 |
|  | PBM | Control | .253 |
|  |  | CM | .876 |
|  |  | CM+PBM | .661 |
|  | CM+PBM | Control | .121 |
|  |  | CM | .553 |
|  |  | PBM | .661 |
| BS.Day16 | Control | CM | .011 |
|  |  | PBM | .001 |
|  |  | CM+PBM | .001 |
|  | CM | Control | .011 |
|  |  | PBM | .248 |
|  |  | CM+PBM | .195 |
|  | PBM | Control | .001 |
|  |  | CM | .248 |
|  |  | CM+PBM | .883 |
|  | CM+PBM | Control | .001 |
|  |  | CM | .195 |
|  |  | PBM | .883 |
| Wei.Day0 | Control | CM | .841 |
|  |  | PBM | .711 |
|  |  | CM+PBM | .075 |
|  | CM | Control | .841 |
|  |  | PBM | .569 |
|  |  | CM+PBM | .051 |
|  | PBM | Control | .711 |
|  |  | CM | .569 |
|  |  | CM+PBM | .149 |
|  | CM+PBM | Control | .075 |
|  |  | CM | .051 |
|  |  | PBM | .149 |
| Wei.Day16 | Control | CM | .880 |
|  |  | PBM | .134 |
|  |  | CM+PBM | .004 |
|  | CM | Control | .880 |
|  |  | PBM | .174 |
|  |  | CM+PBM | .006 |
|  | PBM | Control | .134 |
|  |  | CM | .174 |
|  |  | CM+PBM | .108 |
|  | CM+PBM | Control | .004 |
|  |  | CM | .006 |
|  |  | PBM | .108 |

**T.TEST**

**Groups = Control**

| **Paired Samples Statistics^a^** | | | | | |
| --- | --- | --- | --- | --- | --- |
|  | | Mean | N | Std. Deviation | Std. Error Mean |
| Pair 1 | BS.Day0 | 370.0000 | 6 | 28.39718 | 11.59310 |
|  | BS.Day16 | 521.1667 | 6 | 46.50771 | 18.98669 |
| Pair 2 | Wei.Day0 | 194.8333 | 6 | 26.23293 | 10.70955 |
|  | Wei.Day16 | 156.6667 | 6 | 24.36117 | 9.94541 |
| a. Groups = Control | | | | | |

| **Paired Samples Test^a^** | | | | | | | | | |
| --- | --- | --- | --- | --- | --- | --- | --- | --- | --- |
|  | | Paired Differences | | | | | t | df | Sig. (2-tailed) |
|  |  | Mean | Std. Deviation | Std. Error Mean | 95% Confidence Interval of the Difference | |  |  |  |
|  |  |  |  |  | Lower | Upper |  |  |  |
| Pair 1 | BS.Day0 - BS.Day16 | -151.16667 | 45.92784 | 18.74996 | -199.36498 | -102.96835 | -8.062 | 5 | .000 |
| Pair 2 | Wei.Day0 - Wei.Day16 | 38.16667 | 3.48807 | 1.42400 | 34.50616 | 41.82718 | 26.802 | 5 | .000 |
| a. Groups = Control | | | | | | | | | |

**Groups = CM**

| **Paired Samples Statistics^a^** | | | | | |
| --- | --- | --- | --- | --- | --- |
|  | | Mean | N | Std. Deviation | Std. Error Mean |
| Pair 1 | BS.Day0 | 410.8333 | 6 | 70.21515 | 28.66521 |
|  | BS.Day16 | 412.1667 | 6 | 69.52530 | 28.38358 |
| Pair 2 | Wei.Day0 | 191.6667 | 6 | 16.46410 | 6.72144 |
|  | Wei.Day16 | 159.0000 | 6 | 15.94992 | 6.51153 |
| a. Groups = CM | | | | | |

| **Paired Samples Test^a^** | | | | | | | | | |
| --- | --- | --- | --- | --- | --- | --- | --- | --- | --- |
|  | | Paired Differences | | | | | t | df | Sig. (2-tailed) |
|  |  | Mean | Std. Deviation | Std. Error Mean | 95% Confidence Interval of the Difference | |  |  |  |
|  |  |  |  |  | Lower | Upper |  |  |  |
| Pair 1 | BS.Day0 - BS.Day16 | -1.33333 | 6.43946 | 2.62890 | -8.09113 | 5.42447 | -.507 | 5 | .634 |
| Pair 2 | Wei.Day0 - Wei.Day16 | 32.66667 | 3.07679 | 1.25610 | 29.43777 | 35.89556 | 26.006 | 5 | .000 |
| a. Groups = CM | | | | | | | | | |

**Groups = PBM**

| **Paired Samples Statistics^a^** | | | | | |
| --- | --- | --- | --- | --- | --- |
|  | | Mean | N | Std. Deviation | Std. Error Mean |
| Pair 1 | BS.Day0 | 417.1667 | 6 | 82.84061 | 33.81954 |
|  | BS.Day16 | 365.6667 | 6 | 73.29302 | 29.92175 |
| Pair 2 | Wei.Day0 | 200.6667 | 6 | 26.91963 | 10.98989 |
|  | Wei.Day16 | 180.5000 | 6 | 26.99444 | 11.02044 |
| a. Groups = PBM | | | | | |

| **Paired Samples Test^a^** | | | | | | | | | |
| --- | --- | --- | --- | --- | --- | --- | --- | --- | --- |
|  | | Paired Differences | | | | | t | df | Sig. (2-tailed) |
|  |  | Mean | Std. Deviation | Std. Error Mean | 95% Confidence Interval of the Difference | |  |  |  |
|  |  |  |  |  | Lower | Upper |  |  |  |
| Pair 1 | BS.Day0 - BS.Day16 | 51.50000 | 18.45806 | 7.53547 | 32.12945 | 70.87055 | 6.834 | 5 | .001 |
| Pair 2 | Wei.Day0 - Wei.Day16 | 20.16667 | 2.31661 | .94575 | 17.73554 | 22.59780 | 21.323 | 5 | .000 |
| a. Groups = PBM | | | | | | | | | |

**Groups = CM+PBM**

| **Paired Samples Test^a^** | | | | | | | | | |
| --- | --- | --- | --- | --- | --- | --- | --- | --- | --- |
|  | | Paired Differences | | | | | t | df | Sig. (2-tailed) |
|  |  | Mean | Std. Deviation | Std. Error Mean | 95% Confidence Interval of the Difference | |  |  |  |
|  |  |  |  |  | Lower | Upper |  |  |  |
| Pair 1 | BS.Day0 - BS.Day16 | 75.16667 | 10.49603 | 4.28499 | 64.15176 | 86.18158 | 17.542 | 5 | .000 |
| Pair 2 | Wei.Day0 - Wei.Day16 | 17.83333 | 3.60093 | 1.47007 | 14.05439 | 21.61227 | 12.131 | 5 | .000 |
| a. Groups = CM+PBM | | | | | | | | | |

| **Paired Samples Statistics^a^** | | | | | |
| --- | --- | --- | --- | --- | --- |
|  | | Mean | N | Std. Deviation | Std. Error Mean |
| Pair 1 | BS.Day0 | 435.0000 | 6 | 81.81687 | 33.40160 |
|  | BS.Day16 | 359.8333 | 6 | 76.98939 | 31.43079 |
| Pair 2 | Wei.Day0 | 224.0000 | 6 | 34.84250 | 14.22439 |
|  | Wei.Day16 | 206.1667 | 6 | 34.85063 | 14.22771 |
| a. Groups = CM+PBM | | | | | |

Microbe Analysis

| **Case Summaries** | | | |
| --- | --- | --- | --- |
| Groups | | S.areus.Day8 | S.areus.Day16 |
| Control | Mean | 6371.6667 | 5200.0000 |
|  | Std. Deviation | 177.81076 | 653.88072 |
| CM | Mean | 4653.3333 | 878.3333 |
|  | Std. Deviation | 217.40898 | 84.24172 |
| PBM | Mean | 2880.0000 | 308.0000 |
|  | Std. Deviation | 323.97531 | 40.37821 |
| CM+PBM | Mean | 1510.0000 | 185.8333 |
|  | Std. Deviation | 148.45875 | 28.70830 |
| Total | Mean | 3853.7500 | 1643.0417 |
|  | Std. Deviation | 1883.02442 | 2137.05098 |

| **ANOVA** | | | | | | |
| --- | --- | --- | --- | --- | --- | --- |
|  | | Sum of Squares | df | Mean Square | F | Sig. |
| S.areus.Day8 | Between Groups | 80523545.833 | 3 | 26841181.944 | 521.483 | .000 |
|  | Within Groups | 1029416.667 | 20 | 51470.833 |  |  |
|  | Total | 81552962.500 | 23 |  |  |  |
| S.areus.Day16 | Between Groups | 102855142.792 | 3 | 34285047.597 | 313.742 | .000 |
|  | Within Groups | 2185556.167 | 20 | 109277.808 |  |  |
|  | Total | 105040698.958 | 23 |  |  |  |

**Post Hoc Tests**

| Dependent Variable | (I) Groups | (J) Groups | Sig. |
| --- | --- | --- | --- |
|  |  |  |  |
| S.areus.Day8 | Control | CM | .000 |
|  |  | PBM | .000 |
|  |  | CM+PBM | .000 |
|  | CM | Control | .000 |
|  |  | PBM | .000 |
|  |  | CM+PBM | .000 |
|  | PBM | Control | .000 |
|  |  | CM | .000 |
|  |  | CM+PBM | .000 |
|  | CM+PBM | Control | .000 |
|  |  | CM | .000 |
|  |  | PBM | .000 |
| S.areus.Day16 | Control | CM | .000 |
|  |  | PBM | .000 |
|  |  | CM+PBM | .000 |
|  | CM | Control | .000 |
|  |  | PBM | .007 |
|  |  | CM+PBM | .002 |
|  | PBM | Control | .000 |
|  |  | CM | .007 |
|  |  | CM+PBM | .529 |
|  | CM+PBM | Control | .000 |
|  |  | CM | .002 |
|  |  | PBM | .529 |
